# Oral Health and Autism Spectrum Disorders: A Unique Collaboration between Dentistry and Occupational Therapy

**DOI:** 10.3390/ijerph18010135

**Published:** 2020-12-27

**Authors:** Dominique H. Como, Leah I. Stein Duker, José C. Polido, Sharon A. Cermak

**Affiliations:** 1 Mrs. T.H. Chan Division of Occupational Science and Occupational Therapy, Herman Ostrow School of Dentistry, University of Southern California, Los Angeles, CA 90089, USA; lstein@chan.usc.edu (L.I.S.D.); cermak@chan.usc.edu (S.A.C.); 2 Children’s Hospital Los Angeles and Herman Ostrow School of Dentistry, University of Southern California, Los Angeles, CA 90089, USA; JPolido@chla.usc.edu

**Keywords:** autism spectrum disorder, dentistry, interprofessional collaboration, occupational therapy, oral health

## Abstract

Children with autism spectrum disorders (ASD) are at risk for oral health disparities. With the dramatic rise in ASD prevalence to 1 in 54 children, it is likely that an increasing number of dental practitioners will encounter or be asked to treat children with ASD. This paper reviews explanations related to the increasing prevalence of ASD, provides reasons why children with ASD are at increased risk for poor oral health, and discusses unique interprofessional collaborations between dental practitioners and occupational therapists. Occupational therapists and dentists can work together to plan modifications to the dental environment or adapt dental protocols to reduce some of the barriers encountered by those with ASD, provide desensitization strategies before the clinic visit, or help a child with emotional regulation during clinical treatments.

## 1. Introduction

Children with special health care needs (CSHCNs), such as autism spectrum disorders (ASD), are at greater risk for experiencing oral health disparities than the general population [1,2,3]. The prevalence of ASD has risen dramatically to 1 in 54 children [4]. Given the high prevalence of ASD, it is likely that an increasing number of dental practitioners will encounter children with ASD in their practice or will be asked to treat children with ASD. In this paper we review reasons related to the increasing prevalence of ASD, provide reasons why children with ASD are at increased risk for poor oral health, and discuss unique collaborations among occupational therapists and dentists that may enhance care for this population.

## 2. The Rise of ASD

ASD is a neurodevelopmental disorder characterized by deficits in initiating social interactions, maintaining social communication, and a range of restricted repetitive behaviors (RRBs), interests, and activities [5]. Deficits in social interactions and communication include challenges in social-emotional reciprocity, non-verbal communication, and establishing, or even understanding, relationships. RRBs include repetitive mannerisms (hand flapping, rocking), stereotyped behaviors, unusually strong interests, or perseverations. Sensory hypo- or hyper-reactivity to sensory stimuli recently has been added to this category [5]. As the name suggests, ASD is a continuum from mild to severe [6,7,8].

Historically, autism was considered extremely rare. One of the earliest reports, based on behavior questionnaires, interviews, and case records, estimated prevalence to be about 4.5 per 10,000 [9]. However, in the last half-century, there has been a dramatic increase in autism diagnoses. The estimated prevalence of ASD in the United States has been monitored by the Centers for Disease Control and Prevention (CDC) Autism and Developmental Disabilities Monitoring Network (ADDM) since 2000, with increases in rates of ASD with each surveillance. In 2000, the prevalence of ASD was 1 in 150. In 2006, it was 1 in 110, and in 2012 it rose to 1 in 68. In the most recent report, published in 2020 based on the 2016 surveillance year, the rate increased to 1 in 54 [4,10].

Research is exploring several theories to explain the changes in prevalence in ASD over time, including diagnostic-related changes (e.g., changes to the criteria for diagnosis, advancements in diagnostic tools, new policies regarding early diagnosis/intervention) [5,11,12,13,14,15,16,17,18,19,20,21,22,23,24,25,26,27,28] and increased focus on gender, racial and ethnic disparities [29,30,31,32,33,34,35,36,37,38,39]. Other aspects include increased recognition of the role of genetics [40,41,42,43,44,45], prenatal influences [46,47,48,49], changes in stigma [50,51,52,53], parental age [48,54,55], and pollution and other environmental factors [44,45,46,47,49,56,57,58,59,60]. Likely, a combination of factors rather than one single factor contributes to the etiology of ASD.

## 3. Oral Health and Autism Spectrum Disorders

Oral health care is a major public health concern and has been identified as a Leading Health Indicator, which are topic areas determined to be of high-priority for action [61]. Given the high prevalence of ASD, it is likely that an increasing number of dental practitioners will encounter children with ASD in their practice or will be asked to treat children with ASD. Therefore, it is important to consider how the symptomatology and severity of an ASD diagnosis may impact health and how interprofessional approaches might improve care for this population.

### Risk Factors for Oral Health Challenges

Research has identified a number of factors that contribute to poor oral health in children with ASD, including difficulty tolerating home and professional oral care, sensory processing differences, uncooperative behaviors, communication impairments, as well as challenges finding and accessing professional oral care services.

Children with ASD experience challenges with oral care both at home and at the dentist. One study reported that as few as 50% of children with ASD brushed their teeth the recommended twice per day, and up to 61% of parents with children with ASD report that toothbrushing is difficult [62,63]. This may be attributed in part to the fact that up to 90% of children with ASD experience sensory processing differences [64]. In the home, the child may experience difficulty with the taste or texture of toothpaste or the tactile sensation of the toothbrush bristles in the mouth. In the dental office, there may be challenges with the taste or smell of prophylaxis paste or fluoride, the dentist touching their face, bright lights shining in the child’s eyes, the high-pitched sound of dental equipment, and unusual smells [62,65]. These types of sensory sensitivities have been associated with the uncooperative behaviors of children with ASD in the dental office [65,66].

Uncooperative behaviors (e.g., crying, screaming, verbal protests, unwillingness to comply) also may influence the success of the visit. These refusals of treatment require dental practitioners to consider alternative methods, such as the use of restraint, general anesthesia, or even the refusal of the dentist to treat the patient [67]. In a study that analyzed data from a national survey of general dentists, dentists reported that the patient’s behavior was a high level barrier to their willingness to treat CSHCNs [68]. The American Academy of Pediatric Dentistry recommends a number of basic behavior guidance techniques to help a child, including Tell–Show–Do, voice control, nonverbal communication, and positive reinforcement [67]; however, these traditional strategies may not be sufficient for children with ASD.

Impairments in communication may make it difficult for children to express their needs and discomfort. Research suggests that children with ASD who demonstrate impairments in communication often have more unmet health needs than those without communication difficulties [69,70]. Similarly, ASD-related behaviors can serve as a barrier to care in the dental clinic. For example, some children with ASD exhibit repetitive or stereotypic movements, such as rocking or hand flapping, both of which might hinder dental treatment.

In addition, many families of children with ASD experience a difficult time locating dentists willing to treat their child [62,71]. In fact, only 40% of general dental practitioners stated that they would work with children with ASD [72]. This could be due to a multitude of reasons, one of which is the dental practitioners’ perceived ability to work with the population. In a survey of general dentists in the United States, the majority of the respondents reported that their dental education had not prepared them well to work with special needs populations [72,73,74]. Internationally, similar results were found in a survey of dentists in Saudi Arabia (n = 108), with over 75% of the respondents indicating that additional education was required to feel comfortable managing patients with special health care needs [75]. Those dentists who reported feeling more prepared were more likely to “set up their practice so that special needs patients could be treated” [72] (p. 1114). Similar results were found in a study that examined the attitudes and behaviors of general and pediatric dentists’ in relation to their patients with ASD; the more prepared the dentists’ felt, the more likely they were to provide care for this population [76]. Without specialized training, general dental practitioners may not be confident in their abilities to address the very specific needs of the ASD population [73,74,77].

Furthermore, working with children with ASD may require additional effort. A survey of Special Care Dentistry Association Members reported that dental practitioners used an average of approximately six accommodations to facilitate dental visits and address difficulties (i.e., communication, routine, social interactions) presented by some children with ASD [78]. The majority of dental practitioners also reported that the financial compensation was inadequate when treating patients with ASD due to the additional time required to prepare for and complete a visit [78]. Dentists are often only able to bill for services rendered (i.e., oral exam), not the associated costs of additional staff, lengthy treatment time, or extra desensitization visits. Current funding practices de-incentivize practitioners from serving this population.

Due to the high prevalence of the above-mentioned challenges, it is especially important that treating dentists be aware of the possibility of encountering sensory processing differences and uncooperative behaviors, as well as the available strategies to combat them.

## 4. Leveraging the Dental Strategic Initiative to Benefit CSHCNs

Education and continued training are important components in the development of a workforce prepared to serve diverse, vulnerable populations. The Advancing Dental Education in the 21st Century project recommends interprofessional collaboration as a means of strengthening the educational foundation of dental practitioners [79]. Knowing the difficulties that some individuals with special health care needs encounter when seeking oral care in the dental clinic and the increasing prevalence of ASD in the United States and abroad, it is important to explore the potential impact these proposed educational and training strategies will have on preparing dental practitioners to serve populations with special needs.

### 4.1. Suggestions to Address ASD Population Needs

#### 4.1.1. Patient-Centered Model of Care

Although the Advancing Dental Education in the 21st Century project was not specifically designed to address the demands of CSHCNs, such as ASD, many of the suggestions are meaningful options in addressing challenges those with ASD encounter. One of the proposed strategies is to move to a patient-centered model of care. The recommendation is to “increase the time that [dental] students spend in community settings, such as clinics, hospitals and dental groups” and “operate some dental school clinics as patient-centered delivery systems” [79] (p. eS9). This transition to a patient-centered model will expose more general dental practitioners to the diversity of patients they will likely encounter in their practice and provide increased opportunities for hands-on learning experiences, both of which are important when treating children with ASD. Additionally, operating in a patient-centered delivery system would ensure that other health professionals are practicing toward the same primary goal of providing comprehensive patient care [79].

CSHCNs often have complex medical conditions that require integrated care. The medical home model was originally introduced by the American Academy of Pediatrics (AAP) to provide care that facilitates participation between patient, family, clinicians, and medical staff [80]. Team-based care has been shown to be efficacious in prevention, health maintenance, and illness management [81]. In a nationally representative sample, researchers found that care in a patient-centered medical home was significantly associated with higher parent-reported health care quality for CSHCNs, with an increased likelihood of accessing prescriptions and other office-based services [82].

#### 4.1.2. Integrated Educational Programming

Interdisciplinary work does not have to be limited to clinical practice. The benefits of integrating multiple healthcare fields’ educational programming are also noted in the recommendations. The proposed methods of integration include enhancing “dental curricula and support[ing] the integration of oral and systemic health content in the curricula of all health professions programs” [79] (p. eS11). One benefit of implementing an integrative approach to oral health for those with ASD is that it will better prepare dental practitioners to collaborate effectively with other health care professionals. Given the impact of oral care on overall health, it is important that this interdisciplinary collaboration occurs.

#### 4.1.3. Interdisciplinary Collaboration

As previously stated, education and continued training are important components in the development of a workforce that is prepared to work with diverse, largely underserved populations. Interprofessional collaboration may be one way to strengthen the educational foundation of dental practitioners in the clinic. Interdisciplinary collaboration with other fields, such as Occupational Therapy, Behavioral Therapy, Psychology, Special Education, Computer Science, and Informatics, provide different perspectives on care and strategies to increase the probability of success for children with ASD in the dental clinic. These strategies range from providing education about sensory sensitivities and creating a sensory-friendly environment; assisting with the development and use of visual supports and social stories, and/or use of video/virtual technology. These strategies all have, at least, preliminary support for their use for children with ASD [66,83,84,85,86,87,88,89,90,91,92,93].

Sensory-friendly environments can modify the sensory experiences encountered by children with ASD, either adding (e.g., weighted blankets, calming music) or removing (e.g., bright lights, loud noises) sensory stimuli encountered during dental care or in the waiting room to decrease stress and anxiety [66,85,86,94]. Strategies incorporating visual supports may improve cooperation and participation; these may include visual schedules, which are visual representations of a task broken down and sequenced into step-by-step directions, and visual clocks, which are particularly helpful in aiding the child to keep track of time [66,90]. Video tutorials have been developed to improve children with ASD’s toothbrushing technique by providing virtual modeling of target behaviors [91]. Video-modeling of a child successfully undergoing a dental visit and watching a favorite movie as a distractor are possible interventions to improve fear and uncooperative behaviors during dental visits [74]. Interventions based on high-tech alternative and augmentative communication (AAC) techniques have also been found to improve self-care activities and cooperation during dental treatments when utilized as part of a digital media support system as well as to inform the development of personalized information and communication technologies (ICT) app [92,93].

Overall, numerous strategies to enhance oral care for children with ASD, often from interdisciplinary teams, have demonstrated success for children with ASD. However, future research is needed to determine which interventions are best suited for which children, especially due to the heterogenous nature of the symptomology for children with ASD and how it may present in the dental clinic. This highlights the need for interprofessional collaboration, which will benefit all fields involved in improving the lives of children with ASD.

##### Interdisciplinary Collaboration: A Focus on Occupational Therapy

One specific interdisciplinary collaboration that may be beneficial to improve oral care for children with ASD involves integrating the field of occupational therapy into dentistry. Occupational therapy uses everyday life activities to enhance and enable participation in what people need to do, what they want to do, and what they are expected to do [95,96]. One of the main focuses of occupational therapy is activities of daily living (including oral care), and it is one of the most frequently used services among those with ASD [97]. Occupational therapists (OTs) often work with children with ASD in school, in their home, and in community settings where they evaluate factors (e.g., sensory, motor, cognitive, social, and communication) that affect individuals’ skills and participation in everyday life activities [98]. As such, OTs are adept at identifying strengths and challenges while adapting and modifying activities to facilitate participation.

Collaborating with OTs who regularly provide services to children with ASD may facilitate dental practitioners’ understanding of the heterogeneity and symptomology of the ASD population, as well as provide possible strategies to improve oral care to this population. Occupational therapy is a discipline that can aid the child through dental care either in the clinic during treatment (i.e., emotional regulation–the ability to monitor and modulate emotions) or before the clinic visit (i.e., in-home desensitization strategies, social stories). OTs can work with dentists to plan modifications to the dental environment or adapt dental protocols to reduce some of the barriers encountered by those with ASD [66,99].

An example of interdisciplinary collaboration is currently underway at the University of Southern California (USC) between the Chan Division of Occupational Science and Occupational Therapy, the Herman Ostrow School of Dentistry at USC, Children’s Hospital Los Angeles (CHLA) Division of Dentistry, and the mental health program at the USC University Center for Excellence in Developmental Disabilities (UCEDD) based at CHLA. As part of a research study funded by the National Institutes of Health’s National Institute of Dental and Craniofacial Therapy, a multidisciplinary team led by OTs has designed a sensory adapted dental environment that modifies the lighting, sounds, and tactile sensations experienced at the dental clinic to reduce anxiety and increase cooperative behaviors in children with ASD [94]. In addition to the environmental adaptations, the OTs developed a social narrative (“social story”) using real-life photographs of a happy child in the dental office where the child with ASD would be receiving care (e.g., waiting room, hallway, private room) to illustrate what would happen during the visit to the Dental Clinic. The parent was asked to read the story to their child several times before their dental visit to help them prepare for the activities of the visit. This is important because many children with ASD become distressed with new, novel, or unpredictable situations, and social narratives provide the child with behavioral guidance stated in positive terms, such as, “When the dentist looks at my teeth, I will keep my mouth open” [100]. In implementing this protocol, occupational therapy doctoral students work alongside pediatric dental residents in the CHLA Division of Dentistry, where an exchange of knowledge continuously occurs. USC occupational therapists, CHLA dentists, and UCEDD psychologists have collaboratively published several manuscripts and presented at dental, psychology, and occupational therapy, and interdisciplinary autism conferences, e.g., [94,101].

Other collaborative activities at USC include inviting OTs to participate in professional proceedings and grand rounds. For the last ten years, OTs have provided seminars to USC pediatric dental residents, describing the particular challenges children with ASD experience in relation to oral care and strategies that can be utilized by dental practitioners to improve care for this population.

## 5. Conclusions

The prevalence of ASD is increasing, and many individuals with ASD experience oral care challenges related to difficulties in communication, sensory sensitivities, and uncooperative behaviors. As such, there is an increasing need for dentists who are trained to work with this population.

The dental field is currently re-examining ways to improve educational programming, ultimately impacting future service delivery. This is an ideal time to address these issues. One of the most promising approaches is to improve interprofessional collaborations, which will aid in the development of a more competent workforce who are willing and able to work with diverse populations. Collaborating with OTs may provide ASD-specific strategies that dental practitioners’ can add to their existing toolbox to serve the needs of the ASD population and provide client-centered strategies dental practitioners can use to best serve those with ASD best. Ultimately, the more confident dental practitioners feel in their knowledge and abilities, the more likely they are to include individuals with ASD in their practice.
